# i-Factor™ Bone Graft Versus Demineralized Bone Matrix for Single-Level Anterior Cervical Discectomy and Fusion: A Propensity Score-Matched Analysis

**DOI:** 10.3390/jcm15114120

**Published:** 2026-05-26

**Authors:** Dong Hun Kim, Jung-Woo Hur, Jin-Young Kim, Jae-Taek Hong

**Affiliations:** 1 Department of Neurosurgery, Bucheon St. Mary’s Hospital, College of Medicine, The Catholic University of Korea, Seoul 14647, Republic of Korea; 2 Department of Neurosurgery, Eunpyeong St. Mary’s Hospital, College of Medicine, The Catholic University of Korea, Seoul 03312, Republic of Korea

**Keywords:** anterior cervical discectomy and fusion, i-Factor, P-15 peptide, fusion rate, demineralized bone matrix, bone graft substitute, cervical spine, spinal fusion

## Abstract

**Background/Objectives:** i-Factor™ Bone Graft is a composite bone substitute containing P-15 synthetic collagen fragment that has demonstrated noninferiority to local autograft in single-level anterior cervical discectomy and fusion (ACDF); however, direct head-to-head comparisons with demineralized bone matrix (DBM) using contemporary 3D-printed titanium cages are lacking. The aim of this retrospective study was to compare radiographic fusion rates, segmental stability, and clinical outcomes between i-Factor™ and DBM in single-level ACDF, with a particular focus on the early time course of fusion. **Methods:** A retrospective propensity score-matched cohort study was conducted in patients with single-level cervical degenerative disc disease (cervical disc herniation, cervical spondylotic radiculopathy, or cervical spondylotic myelopathy) operated between December 2021 and January 2024 at a single tertiary care hospital. Seventy-six consecutive patients undergoing single-level ACDF with 3D-printed titanium cages were matched 1:1 (i-Factor™ vs. DBM) on age, sex, and operative level. Fusion status was assessed by serial dynamic radiographs at 1, 3, 6, and 12 months and by 3D-CT at 12 months in all patients (with additional CT at earlier timepoints when plain films were equivocal), by two independent spine surgeons blinded to graft type; inter-rater agreement (Cohen’s κ) was computed. **Results:** Mean follow-up was 18.1 months. Fusion rates for i-Factor™ at 3, 6, and 12 months were 94.7%, 100%, and 100%, respectively, compared to 71.1%, 84.2%, and 94.7% for DBM. The differences were statistically significant at 3 months (*p* = 0.047) and 6 months (*p* = 0.012), but not at 12 months (*p* = 0.493). Inter-rater agreement was almost perfect (κ = 0.86–1.00). No adverse reactions or device-related complications were observed. **Conclusions:** In this matched cohort, i-Factor™ was associated with significantly faster fusion than DBM in single-level ACDF, with similar 12-month fusion rates. No adverse reactions were observed, although the sample size is insufficient to exclude rare complications.

## 1. Introduction

Anterior cervical discectomy and fusion (ACDF) is a well-established surgical procedure for treating cervical degenerative disc diseases, including cervical radiculopathy and myelopathy caused by disc herniation or spondylosis [1,2,3]. The primary goal of ACDF is to alleviate pain and neurological deficits by removing the damaged disc material and achieving solid spinal fusion. Fusion rates, which refer to the successful integration of bone graft material to form a solid bone bridge across the affected vertebrae, are a critical measure of surgical success [4,5].

Historically, autologous bone grafts, particularly those harvested from the patient’s iliac crest, have been considered the gold standard for achieving high fusion rates. However, autologous bone grafts are associated with significant drawbacks, including donor site morbidity, increased operative time, and limited availability [6,7]. These challenges have driven the development of alternative bone graft materials that can provide comparable or superior fusion outcomes without the complications associated with autograft harvesting.

Demineralized bone matrix (DBM) has emerged as a widely used bone graft substitute in spinal fusion surgery. DBM is produced by acid extraction of allograft bone, which removes the mineral component while preserving the organic matrix containing growth factors, including bone morphogenetic proteins (BMPs) [8,9]. Although DBM demonstrates osteoinductive and osteoconductive properties, fusion rates vary considerably depending on the specific DBM product and clinical application [10,11,12].

i-Factor™ Bone Graft (Cerapedics Inc., Westminster, CO, USA) represents a novel composite bone substitute material consisting of P-15 synthetic collagen fragment adsorbed onto anorganic bone mineral (ABM) suspended in an inert biocompatible hydrogel carrier [13,14]. The P-15 peptide is a 15-amino acid synthetic fragment that mimics the cell-binding domain of type I collagen, promoting cellular attachment and stimulating osteogenic differentiation [15,16]. This unique mechanism enhances cellular recognition and attachment, which are essential processes for successful bone fusion.

A pivotal, noninferiority study conducted under a US FDA Investigational Device Exemption (IDE) demonstrated the benefits of i-Factor™ compared to local autograft bone in single-level ACDF at 1-year postoperative follow-up [17]. Long-term follow-up of the IDE cohort has subsequently confirmed durable noninferiority at 6 years [18], and contemporary systematic reviews of P-15/i-Factor™ across spine surgery have consistently reported acceleration of fusion kinetics relative to traditional grafts, with safety profiles comparable to autograft [19,20,21]. These findings have stimulated further interest in evaluating i-Factor™ against other commonly used bone graft materials to establish its clinical efficacy and safety comprehensively.

The objective of this study was to compare the fusion rate and clinical outcomes of single-level ACDF using i-Factor™ Bone Graft versus conventional DBM with a minimum 12-month follow-up.

## 2. Materials and Methods

### 2.1. Study Design and Patient Selection

This retrospective propensity score-matched cohort study was conducted at a single tertiary care hospital (Eunpyeong St. Mary’s Hospital, The Catholic University of Korea). The institutional clinical pathway for ACDF was established in accordance with the Declaration of Helsinki and follows the recommendations of the Korean Society of Spine Surgery and the AOSpine Knowledge Forum for the surgical management of degenerative cervical disease. The study protocol was approved by the Institutional Review Board of Eunpyeong St. Mary’s Hospital (IRB No. PC22RISI0047, approved on 19 February 2026). Because all clinical and radiographic data were obtained from existing electronic medical records after the clinical care episode, with no prospective intervention or contact with patients for the purpose of the study, the requirement for individual written informed consent was waived by the IRB. All identifiers were removed before analysis.

The source population comprised consecutive adult patients (aged 18–70 years) operated between December 2021 and January 2024 at our institution for single-level cervical degenerative disc disease refractory to at least 6 weeks of conservative management. Eligible pathological conditions included single-level cervical disc herniation, cervical spondylotic radiculopathy, and cervical spondylotic myelopathy. Inclusion required (i) diagnosis of single-level disease confirmed on MRI, (ii) clinical correlation with the affected level on physical examination, (iii) ACDF as the indicated surgical procedure, and (iv) availability of complete radiographic and clinical follow-up of at least 12 months.

Exclusion criteria comprised: (i) multi-level cervical pathology requiring fusion at more than one level; (ii) cervical pathology of non-degenerative origin, including trauma, primary or metastatic tumor, active spinal infection or osteomyelitis, ossification of the posterior longitudinal ligament (OPLL), rheumatoid involvement of the cervical spine, congenital anomaly, or significant cervical deformity (kyphosis or scoliosis); (iii) prior cervical spine surgery at any level; (iv) active systemic infection or active malignancy; (v) DXA-confirmed osteoporosis (T-score ≤−2.5 at the femoral neck or lumbar spine) or other metabolic bone disease (e.g., osteomalacia, primary or secondary hyperparathyroidism, Paget disease); (vi) current pregnancy or planned pregnancy during follow-up (assessed by routine preoperative pregnancy testing in women of childbearing age); and (vii) follow-up of less than 12 months.

Among 156 consecutive patients meeting the inclusion and exclusion criteria during the study period, propensity scores were calculated using a logistic regression model, with the type of bone substitute (i-Factor™ versus conventional DBM [DBX, Musculoskeletal Transplant Foundation, Edison, NJ, USA]) as the dependent variable and age, sex, and operative level as independent variables. Through propensity score 1:1 matching, a total of 76 patients were enrolled, with 38 patients in each group. Although the propensity model included only age, sex, and operative level, additional variables known to influence cervical fusion—body mass index (BMI), smoking status, diabetes mellitus, osteoporosis treatment, and chronic corticosteroid or non-steroidal anti-inflammatory drug (NSAID) use—were extracted from the medical record and compared post hoc between the matched groups (Table 1). The implications of not including these variables in the matching algorithm itself are addressed in the Limitations.

### 2.2. Surgical Procedure

**Figure 1:**
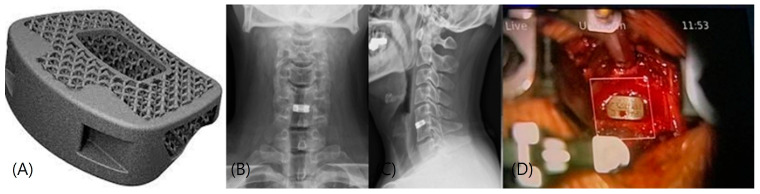
Surgical procedure and implant. (**A**) Stand-alone 3D-printed porous titanium cage. (**B**) Anteroposterior radiograph showing cage position. (**C**) Lateral radiograph demonstrating proper cervical alignment. (**D**) Intraoperative photograph showing the cage filled with i-Factor™ inserted in the disc space. All images are original captures from this study cohort. Representative case: a 53-year-old female with right-sided C5–6 disc herniation and C6 radiculopathy refractory to conservative treatment who underwent single-level C5–6 ACDF using a stand-alone 3D-printed porous titanium cage filled with i-Factor™.

All patients underwent standard single-level ACDF surgery using a stand-alone 3D-printed titanium porous cage (Conduit™ EIT Cellular Tritanium Anterior Cervical Cage, DePuy Synthes, MA, USA) filled with the designated bone substitute (Figure 1). All procedures were performed by a single experienced spine surgeon following a uniform surgical protocol to minimize variability. Cage footprint and height were selected by intraoperative trial fitting based on disc-space anatomy; all cages used a fixed lordotic angle of 4° (manufacturer specification). Cage geometry by group is reported in Table 1.

The surgical technique involved a standard Smith–Robinson approach through an anterior cervical incision. After identification of the target level under fluoroscopic guidance, a complete discectomy was performed including removal of the posterior longitudinal ligament to achieve adequate neural decompression. The cartilaginous endplates were carefully removed while preserving the bony endplates to prevent subsidence. Following neural decompression, the appropriately sized cage filled with the respective bone substitute (1 cc) was inserted under C-arm fluoroscopic guidance. Proper cage positioning was confirmed with intraoperative fluoroscopy.

### 2.3. Clinical Assessment

Clinical outcomes were measured at baseline, 3 months, 6 months, and 12 months postoperatively. Patient-reported outcome measures included the Visual Analogue Scale (VAS) for neck pain and arm pain (0–10 scale; Huskisson [22]) and the Neck Disability Index (NDI; Vernon and Mior [23]). Both instruments are validated and widely used in cervical spine surgery research. Adverse events and complications, including dysphagia, hematoma, infection, and allergic reactions, were recorded throughout the follow-up period.

### 2.4. Radiographic Assessment

**Figure 2:**
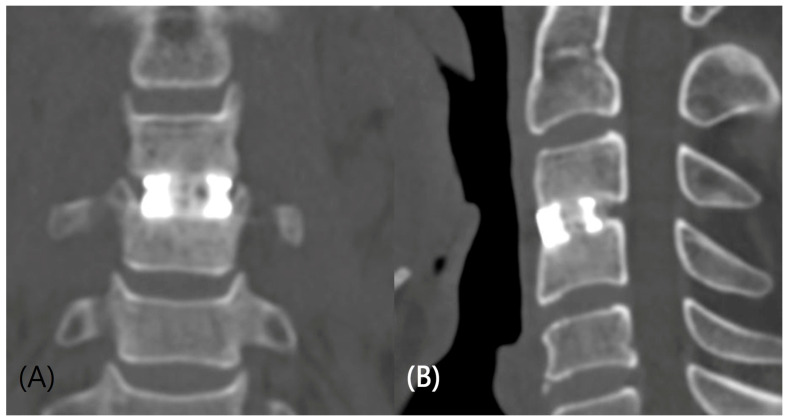
Radiographic assessment of fusion. Coronal (**A**) and sagittal (**B**) reconstructed 3D-CT images at 12 months postoperatively, demonstrating solid bone bridging and complete fusion across the operated disc space in a patient who received i-Factor™ Bone Graft. Original images from this study cohort. Patient: a 46-year-old male who underwent single-level C3−4 ACDF for with C3−4 disc herniation and compressive myelopathy refractory to conservative treatment.

Radiologic evaluation comprised serial plain radiographs and 3D-CT scans. Plain radiographs (anteroposterior, lateral, and dynamic flexion–extension views) were obtained in all patients at 1, 3, 6, and 12 months postoperatively. 3D-CT scans were obtained in all patients at 12 months as the final fusion assessment, and additionally at 3 or 6 months whenever plain radiographs were equivocal for fusion. When CT was available at a given timepoint, fusion status was based on the integrated CT and dynamic radiograph findings; when CT was not available, fusion status was based on dynamic radiographs alone. Fusion status was assessed at 3, 6, and 12 months postoperatively by two independent spine surgeons blinded to the treatment groups (Figure 2). Criteria for solid fusion included: (1) absence of motion (less than 2 degrees of angular motion) on dynamic flexion–extension radiographs, (2) evidence of continuous bone bridging on CT scans (when available), and (3) absence of radiolucent lines around the cage. Disagreements were resolved by consensus. Inter-rater agreement for the binary fusion classification (fused vs. not fused) was quantified by Cohen’s κ at each timepoint. Observed agreement was κ = 0.86 at 3 months, κ = 0.91 at 6 months, and κ = 1.00 at 12 months, all corresponding to almost perfect agreement (Landis & Koch criteria).

Segmental range of motion (ROM) was measured on dynamic lateral radiographs using the Cobb method. Cage subsidence was defined as a decrease of more than 3 mm in segmental height compared to the immediate postoperative measurement. Device-related complications including cage migration and cage breakage were also evaluated.

### 2.5. Statistical Analysis

Statistical analysis was performed using SPSS software (version 25.0, IBM Corp., Armonk, NY, USA). Continuous variables were expressed as mean ± standard deviation and compared using the Student’s *t*-test or Mann–Whitney U test as appropriate. Categorical variables were analyzed using the chi-square test or Fisher’s exact test. Fusion rates between groups were compared at each time point. Inter-rater agreement was assessed using Cohen’s κ. To support interpretation of the observed differences in the context of Type II error, post hoc power was calculated for the three timepoint comparisons of fusion using a two-sided two-proportion test (α = 0.05) with effect size expressed as Cohen’s h; we acknowledge the recognized limitations of post hoc power based on observed effect sizes and present these values for transparency only. A *p*-value of less than 0.05 was considered statistically significant.

## 3. Results

### 3.1. Patient Demographics

A total of 76 patients met the inclusion and exclusion criteria after propensity score 1:1 matching, with 38 patients in the i-Factor™ group and 38 patients in the DBM group. The two groups were well-matched for age (52.4 ± 8.7 vs. 51.8 ± 9.2 years; *p* = 0.842) and sex (male-to-female ratio 1.24 [21:17] vs. 1.11 [20:18]; *p* = 0.821), and balanced across the three operative levels (C4–5, C5–6, C6–7; *p* = 0.954). The mean follow-up period was 18.1 months (range: 12–26 months). Although BMI, smoking status, diabetes mellitus, osteoporosis treatment, and chronic corticosteroid or NSAID use were not included in the propensity model, post hoc comparison demonstrated that these characteristics were also balanced between the matched groups (Table 1). Implant geometry (cage height, lordosis angle, footprint, and anterior–posterior positioning depth) was likewise comparable between the two groups (Table 1).

### 3.2. Fusion Outcomes

Fusion status was evaluated using serial flexion–extension radiographs and 3D-CT scans. The results demonstrated significantly faster time to fusion in the i-Factor™ group compared to the DBM group (Table 2).

At 3 months postoperatively, 36 of 38 patients (94.7%) in the i-Factor™ group achieved solid fusion compared to 27 of 38 patients (71.1%) in the DBM group (*p* = 0.047). By 6 months, all 38 patients (100%) in the i-Factor™ group had achieved solid fusion compared to 32 of 38 patients (84.2%) in the DBM group (*p* = 0.012). At 12 months, fusion rates were 100% in the i-Factor™ group and 94.7% in the DBM group (*p* = 0.493). Inter-rater agreement (Cohen’s κ) for the binary fusion classification was 0.86, 0.91, and 1.00 at 3, 6, and 12 months, respectively, all consistent with almost perfect agreement.

Segmental range of motion analysis showed significantly lower motion in the i-Factor™ group at 3 months postoperatively (1.62° ± 1.21° vs. 3.83° ± 1.62°, *p* = 0.008), indicating earlier stabilization. By 12 months, both groups achieved similar segmental stability with minimal residual motion.

### 3.3. Clinical Outcomes

Clinical outcomes improved significantly in both groups and were maintained until final follow-up. VAS scores for neck and arm pain, as well as NDI scores, showed similar patterns of improvement in both groups with no statistically significant differences at any time point. Both groups demonstrated substantial pain relief and functional improvement following surgery.

### 3.4. Complications

Subsidence rates were similar between the two groups (2/38 [5.3%] in the i-Factor™ group vs. 3/38 [7.9%] in the DBM group, *p* = 0.644), and no device-related complications such as cage migration or breakage were observed. No allergic or other adverse reactions associated with i-Factor™ use were identified during follow-up; however, given the limited sample size (n = 38 per group), the present cohort cannot exclude rare complications with an incidence below approximately 5%. No patients required revision surgery during the follow-up period.

## 4. Discussion

This propensity score-matched comparative study found that, in this single-center cohort, i-Factor™ Bone Graft was associated with significantly faster radiographic fusion than conventional DBM in single-level ACDF. In the i-Factor™ group, 94.7% of patients achieved solid fusion at 3 months compared to 71.1% in the DBM group (*p* = 0.047), and complete fusion was reached by all i-Factor™ patients by 6 months versus 84.2% in the DBM group (*p* = 0.012). At 12 months, fusion rates were 100% and 94.7%, respectively, with no statistically significant difference (*p* = 0.493); however, this comparison was clearly underpowered (post hoc power ≈ 0.52) and should not be interpreted as evidence of equivalence. These findings support the hypothesis that i-Factor™ may accelerate the early phase of bone healing after ACDF, but a definitive causal interpretation cannot be drawn from a retrospective design.

The earlier fusion observed with i-Factor™ is biologically plausible and consistent with its mechanism of action. The P-15 peptide, a synthetic 15-amino acid sequence that replicates the cell-binding domain of type I collagen, facilitates rapid cellular attachment and stimulates osteogenic differentiation through specific integrin-mediated signaling pathways [15,16]. This biomimetic approach provides a consistent and reproducible mechanism for promoting bone formation at the cellular level. Unlike DBM, which relies on residual growth factors that may vary considerably in concentration and bioactivity between different donor lots and commercial preparations, i-Factor™ offers a standardized osteoinductive stimulus [10]. The anorganic bone mineral component provides an osteoconductive scaffold with a three-dimensional architecture that closely resembles natural bone, while the hydrogel carrier ensures optimal handling characteristics and maintains the peptide’s bioactivity at the surgical site [13,14,21].

The clinical significance of achieving early fusion should not be underestimated, as it has important implications for patient outcomes and healthcare resource utilization. Achieving solid fusion sooner may provide earlier symptomatic relief by eliminating painful micromotion at the operated segment, thereby improving patient satisfaction and quality of life during the critical postoperative recovery period. Earlier fusion also reduces the risk of implant-related complications such as cage subsidence, migration, and hardware failure, which are more likely to occur in the presence of persistent pseudarthrosis [24,25]. Furthermore, patients who achieve early fusion may be able to return to normal daily activities and work responsibilities more quickly, which has significant socioeconomic implications [26].

Our findings are consistent with and extend the results of previous studies evaluating i-Factor™ in spinal fusion applications. The pivotal FDA IDE trial conducted by Arnold et al. [17] demonstrated noninferiority of i-Factor™ compared to local autograft in single-level ACDF, with fusion rates exceeding 95% at 12 months and similar improvements in clinical outcome measures. Importantly, that study also reported faster fusion kinetics in the i-Factor™ group at earlier time points [17], which is consistent with our observations. Subsequent follow-up of the IDE cohort has confirmed durable noninferiority at 24 months [27] and at 6 years [18], and recent systematic reviews have synthesized the available data on P-15/i-Factor™ across cervical, lumbar, maxillofacial, and trauma applications, consistently reporting accelerated bone formation with safety comparable to autograft [19,20,21]. The current study extends these findings by providing a direct comparison with DBM, which represents one of the most commonly used bone graft substitutes in contemporary clinical practice [11,12].

No adverse reactions, including allergic responses, inflammatory complications, or ectopic bone formation, were observed in any patient receiving i-Factor™ in this cohort. These observations are consistent with the safety signal reported across larger series and systematic reviews of P-15 peptide-enhanced graft substitutes [19,20]; however, given the size of the present cohort (n = 38 per group), our data cannot exclude rare (1–2%) complications and should not be interpreted as confirming an excellent safety profile in isolation. In contrast to recombinant human bone morphogenetic protein-2 (rhBMP-2), which has been associated with various complications including dysphagia, soft tissue swelling, radiculitis, ectopic bone formation, osteolysis, and potentially increased cancer risk in the anterior cervical spine [28,29], the synthetic nature of the P-15 peptide and the inert characteristics of the hydrogel carrier in i-Factor™ plausibly contribute to a lower risk of inflammatory or immunogenic responses [19,20,21].

The use of stand-alone 3D-printed porous titanium cages in both groups of our study represents a contemporary approach to ACDF that eliminates the need for supplemental anterior plate fixation. These innovative implants feature a highly porous structure with interconnected pores that promote bone ingrowth and osteointegration, potentially enhancing the fusion process [30,31]. The standardized use of these cages across both treatment groups in our study, combined with the comparable cage geometry between groups (Table 1), provides a controlled comparison of the bone graft materials while incorporating modern implant technology.

This study has several limitations. First, the retrospective design inherently carries risks of selection bias and confounding, despite our use of propensity score matching to control for measured covariates. The propensity model included only age, sex, and operative level. Additional variables known to influence cervical fusion—BMI, smoking, diabetes mellitus, osteoporosis treatment, and chronic corticosteroid or NSAID use—were balanced between the matched groups in post hoc comparison (Table 1) but were not used in the matching algorithm itself; their balance therefore reflects the natural similarity of the cohorts rather than a designed adjustment. Other unmeasured confounders, including bone mineral density (DEXA), postoperative compliance with collar wear, and unrecorded medications affecting bone metabolism, may still have influenced our results. Second, although the cohort is adequately powered to detect the large early differences observed at 3 and 6 months (post hoc power 0.84 and 0.95, respectively), it is underpowered for the 12-month comparison (post hoc power ≈ 0.52); a meaningful difference of less than approximately 14 percentage points could not have been reliably detected at that timepoint. The non-significant 12-month result therefore should not be interpreted as evidence of equivalence between the two grafts, and the present sample size is insufficient to detect rare complications (incidence <5%). We acknowledge the recognized limitations of post hoc power calculations and present them only as a transparency aid for interpreting Type II error. Third, the follow-up period may not be sufficient to detect late pseudarthrosis or long-term complications. Fourth, all surgeries were performed by a single surgeon at a single institution, which may limit external validity. Fifth, the study did not include a formal cost-effectiveness analysis. Sixth, although CT was obtained in all patients at 12 months and selectively at earlier timepoints when plain films were equivocal, the non-uniform availability of CT at 3 and 6 months may have introduced minor measurement variability into the early fusion estimates; we mitigated this by adjudicating fusion using the prespecified blinded multi-modality criteria with documented inter-rater agreement. Seventh, this study lacks a control group involving a different pathological condition (e.g., cervical trauma or post-traumatic instability) or a distinct surgical technique (e.g., ACDF with anterior plating, or cervical disc arthroplasty), which would allow broader assessment of i-Factor™’s comparative performance across clinical scenarios. Larger, prospective, multicenter studies with longer follow-up are needed to confirm whether the early fusion advantage observed here translates into durable differences in clinical outcomes, complications, and cost-effectiveness.

## 5. Conclusions

In this propensity score-matched single-center cohort, single-level ACDF using i-Factor™ Bone Graft was associated with significantly earlier radiographic fusion than ACDF using conventional DBM, with comparable clinical improvement and no observed adverse reactions. Final 12-month fusion rates were not significantly different, although this comparison was underpowered. These findings suggest that i-Factor™ may be associated with faster early fusion in single-level ACDF, but a causal advantage and a difference in long-term outcomes cannot be established from a retrospective cohort of this size. Further prospective, randomized controlled trials with larger sample sizes and longer follow-up periods are warranted to confirm these results.

## Figures and Tables

**Table 1:** Patient demographics, baseline characteristics, comorbidities, and implant geometry of the matched cohort.

Parameter	i-Factor™ (n = 38)	DBM (n = 38)	*p* Value
Age (years)	52.4 ± 8.7	51.8 ± 9.2	0.842
Sex (M:F; ratio)	21:17 (1.24)	20:18 (1.11)	0.821
Operative level			0.954
C4–5	6 (15.8%)	7 (18.4%)	
C5–6	19 (50.0%)	18 (47.4%)	
C6–7	13 (34.2%)	13 (34.2%)	
Follow-up (months)	18.3 ± 4.2	17.9 ± 3.8	0.765
BMI (kg/m^2^)	24.6 ± 3.2	24.9 ± 3.5	0.701
Current smoker, n (%)	9 (23.7)	11 (28.9)	0.601
Diabetes mellitus, n (%)	7 (18.4)	8 (21.1)	0.773
Osteoporosis treatment, n (%)	4 (10.5)	5 (13.2)	0.722
Chronic corticosteroid use, n (%)	2 (5.3)	3 (7.9)	0.644
Chronic NSAID use, n (%)	12 (31.6)	14 (36.8)	0.629
Cage height (mm)	6.4 ± 0.9	6.5 ± 0.8	0.612
Cage lordosis angle (°)	4.0 ± 0.0	4.0 ± 0.0	—
Cage footprint (mm × mm)	12 × 16	12 × 16	—
AP positioning depth (mm)	2.1 ± 0.6	2.2 ± 0.7	0.587

Values are presented as mean ± SD or n (%). Abbreviations: AP, anterior–posterior; BMI, body mass index; DBM, demineralized bone matrix; M:F, male-to-female; NSAID, non-steroidal anti-inflammatory drug; SD, standard deviation.

**Table 2:** Fusion status and segmental range of motion at each follow-up timepoint.

Parameter	1 Month	3 Months	6 Months	12 Months
**Fusion status, n (%)**				
i-Factor™	—	36 (94.7)	38 (100)	38 (100)
DBM	—	27 (71.1)	32 (84.2)	36 (94.7)
*p* value	—	0.047 *	0.012 *	0.493
Post hoc power	—	0.84	0.95	0.52
**Segmental ROM (°)**				
i-Factor™	3.57 ± 1.06	1.62 ± 1.21	1.52 ± 1.23	0.13 ± 1.06
DBM	4.35 ± 0.94	3.83 ± 1.62	2.08 ± 1.68	0.28 ± 1.54
*p* value	0.295	0.008 *	0.233	0.794

Values are presented as n (%) or mean ± SD. * *p* < 0.05. Post hoc power was computed using a two-sided two-proportion test (α = 0.05) with effect size expressed as Cohen’s h. Abbreviations: DBM, demineralized bone matrix; ROM, range of motion; SD, standard deviation.

## Data Availability

The data presented in this study are available on request from the corresponding author due to privacy and ethical restrictions.
